# The impact of FDA-cleared molecular solutions for BK polyomavirus quantitation

**DOI:** 10.1128/jcm.00348-24

**Published:** 2025-01-17

**Authors:** Bijal A. Parikh, Neil W. Anderson

**Affiliations:** 1 Department of Pathology and Immunology, Washington University School of Medicine 12275 , St. Louis, Missouri, USA; 2 Department of Pathology, University Hospitals Cleveland Medical Center 114516 , Cleveland, Ohio, USA; Vanderbilt University Medical Center, Nashville, Tennessee, USA

**Keywords:** BKV

## Abstract

Accurate detection and monitoring of BK polyomavirus (BKV) infection is of critical importance in the post-transplant period, guiding treatment decisions that balance the anti-rejection effects of immune suppression with host-protective effects of immune defense. Historically, test methods for BKV have been independently developed by laboratories to address this unmet need. However, these assays can suffer from inconsistencies in analytical variability, which in turn have hindered the establishment of commutable and clinically actionable viral load thresholds for clinical management. As a result, the interpretation of viral load quantitation has not been standardized across transplant centers for the purpose of monitoring patients at highest risk for infection-related complications. In this review, we describe challenges that have historically limited widespread adoption of BKV quantitative testing. We then detail how developments in the field, including optimized amplicon selection, the introduction of an international standard, and the availability of Food and Drug Administration (FDA)-cleared methods, have played a role in harmonization of quantitative BKV measurements in the clinical management of transplant recipients.

## INTRODUCTION

FDA-cleared methods for the detection of BK polyomavirus (BKV) have been heralded as an important advance for the standardization and widespread adoption of monitoring infection in a critical immunosuppressed patient population in their immediate post-transplant period. Complications of BKV infection cannot be prevented through vaccination or prophylaxis, and there are no effective antiviral therapies to mitigate the disease once confirmed. The reduction of immunosuppression to permit natural host defense mechanisms against BKV is the most effective tool clinicians can leverage, but it must be carefully balanced against transplant loss promoted by the same host immune mechanism. Molecular methods are still considered only a surrogate for highly invasive tissue biopsies, yet greater confidence in the predictive ability of nucleic acid testing has shown promise to minimize reliance on such procedures. Historical challenges in establishing actionable thresholds for the timing and degree of immunosuppression reduction to prevent BKV sequela have been attributed to lack of standardized and commutable detection methods. FDA-cleared methods for accurate, precise, and quantitative BKV monitoring have and will continue to emerge to address prior assay limitations and provide additional opportunities to harmonize diagnostic, prognostic, and therapeutic guidelines for this important infection.

## BKV STRUCTURE AND GENOMIC ORGANIZATION

BKV is a ubiquitous virus belonging to the Polyomaviridae family. Virions are non-enveloped and typically between 40 and 45 nm in diameter. The genome of BKV is circular double-stranded DNA (dsDNA) of approximately 5.3 kilobases (1). A unique feature of polyomavirus genomes is their bidirectional promoter, allowing for transcription of early (large and small tumor antigens, LTAg, and sTAg) and late (VP1, VP2, and VP3 and agnoprotein) genes. BKV comprises four major subtypes (I, II, III, and IV) and additional subgroups based on genomic variation. Subtype I is the most common worldwide (80%) and is divided into subgroups Ia, Ib1, Ib2, and Ic, whereas BKV subtype IV is the second-most common (15%) (2). Regions of VP1 provide sufficient diversity for successful typing; thus, it is equally important to avoid this typing region when developing subtype-agnostic molecular detection assays. Unique to the virology of BKV is an extremely high mutation rate, which is reported to be higher than that of any other dsDNA virus and more similar to that of RNA viruses (3). This allows the virus to evade the immune system and further poses challenges to molecular assay design.

## EPIDEMIOLOGY AND COMPLICATIONS OF BKV

Seroprevalence estimates suggest that up to 90% of individuals by age 10 have been infected with BKV at some point in their life (4). Primary infections during childhood are acquired through oral or respiratory routes, with most individuals manifesting with asymptomatic or a very mild respiratory illness. BKV quickly establishes latency in renal tubular epithelial cells and can cause asymptomatic but intermittent shedding in immunocompetent individuals who may demonstrate urine viral load levels of greater than 1,000 copies/mL (5). Since the virions are nonenveloped, they are remarkably stable in the environment, which likely contributes to the relative ubiquity of BKV. Reactivation from latency in immunocompromised hosts can lead to serious complications, including renal graft failure following renal solid organ transplantation (SOT) or hemorrhagic cystitis (HC) following bone marrow or hematopoietic stem cell transplantation (HSCT). Interestingly, those undergoing SOT receiving organs other than kidneys rarely experience BKV reactivation (6), suggesting that both immunosuppression and renal intrinsic factors are important pathogenic drivers. Reactivation of BKV is typically controlled by the host CD8+ T-cell response (7). These cytotoxic mediators function to eliminate infected cells, produce antiviral cytokines, and support long-term immune memory. Following HSCT or renal SOT, immunocompromised individuals require suppression of T-cell function to prevent graft rejection. However, the dampening of this immune response must be balanced with the possibility of iatrogenic BKV reactivation and associated complications. Conversely, addressing the degree of immunosuppression is often the first-line management in patients with BKV reactivation.

## PATHOLOGY AND LABORATORY FEATURES OF BKV INFECTION FOLLOWING TRANSPLANT

After kidney transplant, BKV reactivation presents as a sequence of pathology and laboratory findings supporting disease progression. Specifically, 30%–60% of recipients develop BKV DNA in the urine (DNAuria), with 10%–20% of those individuals demonstrating virus in the plasma (DNAemia) and 5%–10% of those individuals developing BK virus-associated nephropathy (BKVAN) (8). Progression between these stages occurs over the course of weeks to months. BKVAN, if it were to occur, typically manifests within 2 years but more often 3–9 months following renal transplantation (9). Clinically, patients can be asymptomatic or show an increase in creatinine concentrations, proteinuria, and even hematuria, which can also be interpreted as either acute rejection or drug toxicity. Urine cytology analysis can be performed for determining the presence of shed uroepithelial cells infected with BKV, commonly referred to as “decoy” cells. However, the reported sensitivity and specificity of decoy cells as a marker for progression to BKVAN is lower (66.7% and 88.6%, respectively) than that of immunohistochemical (IHC) staining (85.7% and 92.3%, respectively) or the presence of BKV DNAemia (96.3% and 90.3%, respectively) (10). During active BKV nephritis, infected tubular epithelial cells within the kidney parenchyma can be visualized using renal biopsy and histologic examination. IHC staining using antibodies against the closely related SV40 polyomavirus can further identify cells with viral inclusions as “polyomavirus infected” (11). This indirect approach is taken due to the more ready availability of the SV40 IHC antibody in comparison to BKV-specific IHC.

Regardless of the IHC approach, histologic examination requires an invasive renal biopsy. Hence, monitoring of markers of viral replication in more readily obtained specimens is of great utility. There is generally a stepwise progression from initial DNAuria to subsequent DNAemia. However, plasma BKV may rarely be detected as an isolated finding and may develop through isolated leukocyte reactivation (12). Other considerations in the differential diagnosis of viral nephropathy include infections with adenovirus, JC polyomavirus (JCV), cytomegalovirus (CMV), human herpesvirus-6 and −7, and parvovirus B19 (13).

Following HSCT, recipients are at highest risk for BKV-associated HC (BKV HC) between 2 and 8 weeks after transplant (14). Although the reported incidence varies greatly, the literature suggests that between 5% and 40% of HSCT recipients will progress to BKV HC (15). Individuals with BKV HC present with painful hematuria due to inflammation of the urinary bladder mucosa (16). The differential diagnosis of HC following HSCT includes infection with adenovirus, herpes simplex virus (HSV), JCV, and CMV. Prophylaxis and treatment primarily involve hydration to dilute the urine and reduce bladder irritation and bladder irrigation to remove blood clots and ease pain (14). Guidelines have not been strictly established for correlating BKV load in plasma or urine to the development of HC. Thus, the incidence of BKV DNAuria has been reported to occur in 50%–100% of HSCT recipients. Individual studies have shown that recipients with DNAuria >10^9^ copies/mL are at a higher risk of BKV HC and typically detected 2 weeks prior to HC (17). Earlier studies demonstrated that lower cutoffs of 10^6^ copies/mL are more sensitive in predicting HC (18). These conflicting cutoffs may be due to the lack of standardized methods to quantify BKV DNAuria across studies. In contrast, BKV DNAemia of 1,000 to 10,000 copies/mL may be a more specific predictor of BKV HC development (14), but again lacks sufficient predictive value to be included in universal screening guidelines following HSCT.

## GUIDELINES FOR BKV MONITORING FOLLOWING RENAL TRANSPLANTATION

In 2019, The American Society of Transplantation Infectious Diseases Community of Practice (AST-IDCOP) summarized recommendations for BKV monitoring in renal transplant patients (19). More recently, The Transplantation Society (TTS) reviewed international guidelines on the approach to BKV monitoring (6), with both groups largely harmonized in their recommendations. Both guidelines recognize the limitations of qualitative DNA testing since quantitative testing in urine and plasma is required to differentiate between low-level and high-level viral loads. While neither source provides clear cutoffs for urine screening or monitoring, DNAuria may be used in resource-limited setting or when plasma testing is either not available or not the standard of care. In those situations, plasma viral load testing should be initiated if DNAuria is found to exceed 10 million copies/mL urine. The guidelines further define plasma BKV loads above 1,000 copies/mL persisting for greater than 2 weeks as probable nephropathy and above 10,000 copies/mL in a single sample as presumptive nephropathy. Renal biopsy is still the gold standard for the diagnosis of BKVAN, but due to its higher sensitivity and ease of collection, plasma DNA is the preferred surrogate measure. Both groups also acknowledge that a renal biopsy may not always detect BKV during the early stages of infection or when the disease is resolving and recommend interpreting biopsy results along with clinical data and quantitative plasma BKV testing. Since most BKVAN cases occur within the first 2 years after transplantation, frequent screening during this period is critical. The recommended frequency of testing is monthly until 9 months post-transplant, followed by every 3 months until 2 years post-transplant. AST-IDCOP guidelines lengthen screening duration to 3 years for children. TTS guidelines recommend extended screening for patients who have received combined SOT (renal plus another organ) with testing every 3 months up to 3 years post-transplant. Both urine and plasma specimen types are considered acceptable for monitoring, with plasma levels providing a higher predictive value for BKVAN. Urine specimens are less invasive to collect, but can suffer from higher variability, may fall outside the upper limit of certain assay dynamic ranges, and can be affected by physiological changes in urine composition. Therefore, consensus recommendations favor routine screening of kidney transplant recipients for BKV DNAemia using plasma samples. For recipients with sustained DNAemia above 1,000 copies/mL, guidelines recommend monitoring BKV plasma DNA every 2–4 weeks to assess the treatment response. Ultimately, flexibility exists to allow individual transplant centers to develop and validate their own monitoring schedule and actionable cutoffs based on the methods available to quantify BKV. This flexibility has historically been necessitated due to the lack of standardized methods allowing harmonization of viral load cutoffs.

## CHALLENGES WITH BKV MOLECULAR TESTING

Universal monitoring guidelines for BKV following transplantation have been limited due to several analytical factors, including diversity of acceptable specimen types, viral fragmentation, commutability, representative calibration standards, effects of nucleic acid extraction and purification methods, viral sequence variations, and the availability of harmonized quantitative methods. In fact, quantitation differences between laboratories engaged in multicenter studies can exceed the generally accepted variation of 0.5 log10 copies/mL (20, 21).

Upon comparing viral loads between extracted and unextracted specimens, the majority of detectable BKV DNA in urine is found to be cell-free rather than encapsulated within virions (22). However, comparing different urine treatment protocols and DNA extraction methods demonstrates how the presence of cells impacts the accuracy of viral load measurements as cell-associated BKV can dramatically increase the observed titer levels (23). Intracellular BKV in urine may not reflect reactivation as accurately as cell-free virus quantitation. Additionally, as 15%–40% of healthy individuals can intermittently shed BKV in the urine (24), these combined impacts on the specificity of urine alone in supporting a diagnosis of BKVAN are limited. While more specific than urine, plasma samples require centrifugation of cells prior to analysis; thus, the detected DNA is either encapsulated or virion-free. Studies employing DNAse digestion of plasma specimens demonstrated that >90% of virus was not protected within viral particles (25). A critical preanalytical consideration in interpreting plasma viral loads includes a delay of centrifugation beyond 24 hours post-collection, leading to potential lysis of infected lymphocytes and an inadvertent elevation of the measured viral loads. Furthermore, prolonged contact with whole blood could also lead to degradation of nonencapsulated DNA, exposing the nucleic acid to nucleases for a longer duration, and combined with cellular release can lead to unpredictable effects on plasma DNA quantitation. Interestingly, even when all other variables can be controlled by the use of standard reference materials, the sample matrix (urine vs plasma) can also have a significant effect on quantitation, through yet unclear mechanisms (26). Thus, in addition to specifying the instrumentation, assay design, and incorporation of quantitative standards, BKV monitoring guidelines benefit from referencing methods that describe strict specimen-specific handling and processing workflows.

Encapsulated BKV comprises whole full-length genomes, whereas virion-free DNA in urine and/or plasma can be isolated as shorter pieces or fragments. Viral fragmentation in plasma may be due to the action of extracellular nucleases, the host immune response against circulating DNA, or as a consequence of viral lysis of infected cells with partially replicated genomes. While the origins of these fragmented genomes are not clear, this phenomenon can also be seen for other DNA viruses, including CMV and Epstein–Barr virus (EBV) (27). Regardless, the fragmented nature of BKV DNA can contribute to variability in viral load measurements across different assays. Assays that target larger amplicons might underestimate the true viral load because the fragmented DNA may not contain enough intact copies of the target sequence. A recent study specifically investigated this impact through a series of BKV quantitative molecular tests targeting the LTAg gene with varying amplicon lengths of 88, 133, and 239 bp (25). They showed that the assay detecting the shortest amplicon of 88 bp was consistently more sensitive in identifying samples with low-level BKV. The 88-bp amplicon assay yielded about five times more (0.7 log10) copies/mL of BKV than either of the larger amplicon assays. At higher viral loads, near clinical decision cutoffs of 1,000 and 10,000 copies/mL, the shorter amplicon assay detected BKV with a higher quantitative result, suggesting that assays using larger amplicons may fail to reach a clinical decision threshold simply due to the amplicon size. Finally, the 88-bp assay detected BKV DNA in a subset of samples that were found to be undetectable by the assays with longer amplicons. As a consequence of BKV fragmentation on interassay variability, current approaches to monitor BKV loads should ideally utilize short amplicons optimized to maximize both the sensitivity and specificity. Current guidelines do not specify what amplicon size to use, and commercial manufacturers do not readily provide detailed information on amplicons.

## STANDARDIZATION OF BKV QUANTITATIVE ASSAYS

Commutability, in the context of viral load standardization, refers to the extent to which a reference material behaves like patient samples and is consistent across different assays. This material can be utilized to calibrate quantitative assays such that patient results are comparable from assay to assay. A perfectly commutable standard will show a consistent quantitative relationship with clinical samples across various assay platforms. However, commutability through a common standard can be difficult to achieve under certain conditions. For example, matrix effects (i.e., urine vs plasma) may influence quantitation results as clinical samples may contain a variety of assay inhibitors not found in the calibration suspension. Variations in sample preparation, primers/probes, amplification conditions, and detection instrumentation may affect assay performance. Thus, even disparate assays calibrated to a primary standard or a derived secondary standard may result in very different viral loads for identical clinical specimens. To compound such challenges, differences in standards can worsen discrepancies between assays, impeding efforts to establish universal thresholds for clinical decision-making (17).

The development and deployment of the first World Health Organization (WHO) International Standard (IS) for BKV in late 2015 was intended to minimize interassay variability among testing platforms when assessing identical proficiency testing challenges (20, 28). The lack of a common calibrator was considered to be a major contributing factor to this variability, and therefore the WHO undertook a multicenter study to evaluate candidate materials for a BKV IS to serve as this calibrator. The study involved 33 laboratories from 15 countries that used a variety of molecular assays and methodologies (29). The two candidate materials evaluated included a lyophilized whole BKV virus preparation and a BKV DNA plasmid construct. Ultimately, the lyophilized preparation was selected as it more closely resembles clinical samples, requiring both extraction and amplification. The calibrator is a cell culture isolate, which was originally from a urine sample of a HSCT recipient diagnosed with BKV HC. A cell culture source for a BKV standard provides a scalable and consistent method for producing large quantities of BKV at an appropriate titer (29). Clinical sources can be limiting and can show batch-to-batch variability and be ethically challenging and practically infeasible to source, making them less useful for large-scale production of an IS. While not identical to the form of BK virus found in clinical specimens (partly due matrix effects, fragmentation, and stability), cell culture-derived BKV preparations are similar enough to the native virus and do not need to be chemically inactivated.

Following a multicenter analysis, this cell culture-derived material was established as the first WHO IS for BKV DNA and assigned a potency value of 7.2 log10 IU/mL. Since the WHO IS was intended to serve as a primary calibrant for BKV molecular assays, methods calibrated on the standard can report results in international units (IU/mL) rather than copies/mL. It is important to note, however, that the various assays used to generate the consensus potency value ranged from 5.69 to 8.33 log10 copies/mL, a difference of nearly 1,000-fold. This underscores the idea that identical testing materials can perform very differently across sites and methods due to factors including automated versus manual extraction, identity and size of viral target regions, and differences in instrumentation to detect amplification.

Subsequently, studies using digital PCR (dPCR) (30) and next-generation sequencing (31) revealed that the WHO BKV IS was heterogeneous, harboring subpopulations of BKV with significant genetic variations, including deletions in the LTAg region. Secondary standards are more practical for manufacturers to obtain and distribute and reflect the quantitation assigned to the IS. However, based on what is known about the limitations of the first WHO BKV IS, calibration of secondary standards must be performed outside of the LTAg region since this could otherwise lead to significant underestimation of viral load. Additional recommendations in establishing secondary standards include avoiding highly variable regions, the use of dPCR to confirm quantitative values (30), and the normalization to IU/mL. Commutability of standards is typically investigated across a wide array of in-use platforms and, when possible, whole-virus based controls used to accurately mirror extraction and purification workflows.

As an alternative to the WHO BK IS, the National Institute of Standards and Technology (NIST) has also developed a quantitative BKV standard (NIST BKV). To generate this standard, the entire BKV genome (subtype I) was cloned into a plasmid vector, biologically amplified in *Escherichia coli*, purified, and quantified by dPCR to provide genome copy numbers per fixed volume (32). While viral DNA cannot represent the complexity and heterogeneity encountered in clinical samples, the uniform material provides a reproducible standard with which to compare the analytical variation largely independently of the extraction method. As a consequence, genomic deletions and duplications do not affect detection resulting from incomplete primer and/or probe binding. Importantly, the choice of the calibration standard was recently shown to significantly affect variability among six laboratory-developed tests (LDTs) (26) and between five LDTs compared to an FDA-cleared assay (21). There are no clear recommendations of which standards to employ for BKV quantification. However, if alignment to IUs is desired, then WHO IS must be used. Despite significant advances in the promotion of assay commutability through standardized calibrants, persistent challenges remain for commutability.

## EXTRACTION METHODOLOGY

The choice of extraction and purification methods can significantly influence the quantitation of BKV DNA. Different extraction techniques exhibit varying efficiencies in recovering viral DNA, particularly of fragmented DNA (33), the predominant form of BKV DNA in blood. The efficiency of extraction, or the percentage of DNA recovered from the sample, influences the amount of the template available for PCR amplification. Thus, more efficient extraction methods will result in higher measured BKV viral loads, even when analyzing the same sample. Studies comparing automated and manual extraction methods show that automated systems tend to be better at recovering higher and consistent DNA quantities. As an example, automated extraction using the BioMerieux NucliSENS EasyMAG system achieved higher viral recovery from both urine and plasma specimens across a wide dynamic range, compared with the manual Qiagen QIAamp DNA Blood Mini Kit (34). Carry-over contamination from specimens with very high viral loads to negative samples can also lead to unintended false-positive results. Routine review of positivity rates and evaluation for carry-over are important quality assessment tools during and following assay validation and verification. In addition to the advantages of automated extraction methods in reducing the risk of contamination, they can provide higher throughput and increased precision. However, increased cost, increased sample volume requirements, and potential for technical issues requiring resolution by field service engineers can limit widespread adoption. Among automated platforms, magnetic bead-based technology for extraction and purification of BKV from urine was shown to be superior to a membrane-based method, likely due to more effective removal of PCR inhibitors (35).

## GENETIC VARIABILITY

Genetic variability in BKV can significantly impact the performance of quantitative assays assessing the viral load. Specifically, failure to include variation across subtypes into primer design can lead to significant underestimation of viral load, as was demonstrated across seven different real-time PCR assays for BKV subtypes III and IV (36, 37). Even within BKV subtypes, subgroup differences have been demonstrated to significantly impact quantitative variability by over tenfold (38). Additional studies have shown that detection of VP1 or LTAg alone has inferior sensitivity compared to an assay targeting VP2/VP3 (39). The VP2 and VP3 genes of BKV are highly stable (40), whereas significant sequence differences are seen for both VP1 and LTAg (36). Increased variability in VP1 has also been reported in patients with BK nephropathy (41). Differences in amplicon length or extraction methods may have accounted for some of the observed effects, so a contemporary study described the impact of BKV gene targets under identical extraction conditions (42). While the amplicon sizes were not reported, the study compared three methods (one commercial and two LDTs) that targeted either LTAg alone, VP1 and LTAg, or VP2 and VP3. Although differences in sensitivity, percent agreement, dynamic range, and variability at low viral loads were observed across clinical samples tested on all three assays, the assay targeting VP2 and VP3 (artus BK virus kit) was the most sensitive (limit of detection [LoD] of 28 copies/mL), provided the best dynamic range and was very precise at low viral loads. Strategies to mitigate the impact of sequence variation include the use of degenerate primers, allowing for a certain degree of mismatch, targeting more than a single region of the genome and ensuring high conservation among subtypes, and regular monitoring of assay performance against varied reference materials and locally circulating subtypes. The performance of assays calibrated against monotypic reference materials (subtype Ia) may potentially benefit from the use of mixed-patient standards that reduce the impact of genomic variation (36). While monitoring for evolving sequence variations may be technically challenging for individual labs to perform, the availability of commercial platforms can shift some of this responsibility back to the manufacturers.

## FDA-CLEARED COMMERCIAL TESTING FOR BKV

Ultimately the availability and broad adoption of FDA-cleared assays allows for a standardization of all testing components, an approach to commutability even more effective than the adoption of common calibrants. This, unfortunately, has historically been an unmet need in BKV testing. To date, only Roche Diagnostics has received authorization from the FDA to market BKV testing for quantitative determination of viral load in plasma (43) and urine (44) from transplant recipients. The approval was initially granted for the cobas 6800/8800 Systems, which can manage up to 384 or 960 tests per 8-hour shift, respectively. Since then, the Roche cobas 5800, a smaller instrument capable of 144 tests per 8-hour shift, has also been approved (45). The assay leverages a magnetic bead-based technology for extraction and a dual-target real-time PCR method, amplifying and detecting conserved regions of VP2 and sTAg with labeled hydrolysis probes. A spike-in control is added to each sample to monitor the extraction efficiency. The master mix uses dUTP and AmpErase to minimize the effects of amplicon cross-contamination. Reagents can be maintained on board the instrument for up to 30 days. In addition to fully automated sample preparation and amplification, automated data management validates runs based on passing controls (high positive, low positive, and negative) and assigns qualitative (detected and not detected) and quantitative (below the lower limit of quantitation (LoQ), above the upper LoQ, or within the assay linear range) results. The linear range for plasma samples is 21.5 IU/mL to 10^8^ IU/mL, and the range for urine samples is 200 IU/mL to 10^8^ IU/mL. Across subgroups Ia and Ic and subtypes I, III, and IV, the LoD was equivalent to the lower LoQ. For urine, the LoD across BKV genotypes was 12.2 IU/mL, exhibiting some quantitative variability between the lower LoQ and LoD.

In equivalency studies submitted to the FDA (43), the cobas BKV assay for plasma samples demonstrated a negative percent agreement (NPA) of 100% and a positive percent agreement (PPA) ranging from 67% to 100%, depending on the threshold bins applied. Unfortunately, limited information about the comparator LDT method, including amplicon size and genetic target(s), precludes the possibility of straightforward interpretation of the analysis. Nevertheless, the observed discordance could largely be explained by the slight negative bias of the cobas assay (−0.357 log10 IU/mL). The NPA for urine samples was 100%, with a PPA ranging from 93.9% to 99.5%. The improved PPA of urine compared with plasma can be attributed to the slight positive bias between methods (0.231 log10 IU/mL). Even though both methods were calibrated to the WHO BKV IS, the influence of undisclosed assay variables previously described likely contributed to observed biases.

In a prior study funded by Roche Diagnostics, Fritzsche et al. compared the cobas omni channel to the Altona RealStar assay (46). Primers and probes were based on the LTAg region and thus unlike the current commercially marketed assay. As a direct consequence of referencing an imperfect calibrator in which approximately 80% of the WHO BKV IS lacks the LTAg target of the assay, the obtained IU/mL required adjustment by a factor of 4 to mitigate over-quantitation of patient-derived virus. The authors found that results were generally equivalent to the more labor-intensive comparator method; however, this was the first published demonstration that the fully automated Roche cobas 6800 system was effective in quantitation of BKV from both urine and plasma specimens.

The first description of the performance characteristics of the FDA-cleared assay was provided by Palavecino et al. (47). The authors compared the results of the cobas BKV assay with a LDT using Diasorin analyte-specific reagents. Similar to the studies described in the Roche cobas BKV package insert, plasma BKV quantitation showed a slight negative bias (−0.58 log10 IU/mL), and urine quantitation demonstrated a slight positive bias (0.21 log10 IU/mL) with the FDA-cleared assay. Although these findings highlight the importance of monitoring patients using consistent methods over time, the fixed bias is not believed to have a significant clinal impact if labs were to transition to the automated method.

Recently, a multicenter study across five European sites described the analytical performance of the FDA-cleared cobas BKV assay when compared against various LDT platforms (21). Analytical panels were diluted in BKV-DNA-negative human EDTA plasma at 1,000, 6,000, and 10,000 IU/mL using the WHO BKV IS and the NIST BKV and distributed for accuracy and precision analyses. These levels were chosen to interrogate the performance at the clinical decision thresholds previously defined. In contrast to the LDTs, the cobas BKV assays demonstrated significantly lower variability, regardless of the reference material used. However, when the cobas assays were assessed with the NIST standard, values were reproducibly lower (−0.307 to −0.362 log10 IU/mL) across all five sites, suggesting an underestimation bias for assays calibrated to the WHO BKV IS, attributed to the standard’s intrinsic heterogeneity (30, 31). When assessed against clinical samples, the cobas BKV assay reported quantitative values for a greater proportion of samples previously tested on LDTs, a consequence of the assay’s extended dynamic range. This study demonstrates that although a high degree of *interlaboratory* variability is expected among the LDTs across sites, the clinically significant *intralaboratory* imprecision of LDTs was not similarly observed using the cobas method. The authors conclude that although current guidelines recommend that patients should be monitored for viral loads by the same assay in the same laboratory, standardization of assay calibrators only partially mitigates the variability, providing further justification for the implementation of standardized assay systems.

While not yet approved by the FDA for marketing within the United States, the performance of the Alinity m BKV assay was recently compared against the several established methods, including the Altona RealStar assay, ELITech MGB Alert BKV LDT, and cobas FDA-cleared BKV assay (48). The Alinity m BKV assay is run on the Alinity m analyzer, which is automated and can perform either continuous or random-access testing. Similar to the cobas assay, the Alinity m BKV assay uses dual targets, one within VP2/3 and the other in sTAg (49). This study demonstrated that clinical plasma specimens cotreated well to both Altona RealStar BKV and Roche cobas BKV assays with R^2^ values of >0.94 and an average bias of −0.47 and −0.31 log10 IU/mL, respectively. Any differences were attributed to assay design, including the extraction and calibration methods. Comparison of urine BKV quantitation was only performed for the ELITech BKV assay and again performed favorably with additional detection of some prior negatives, likely due to the dual-target approach of the Alinity assay.

## CONCLUDING COMMENTS

**Fig 1:**
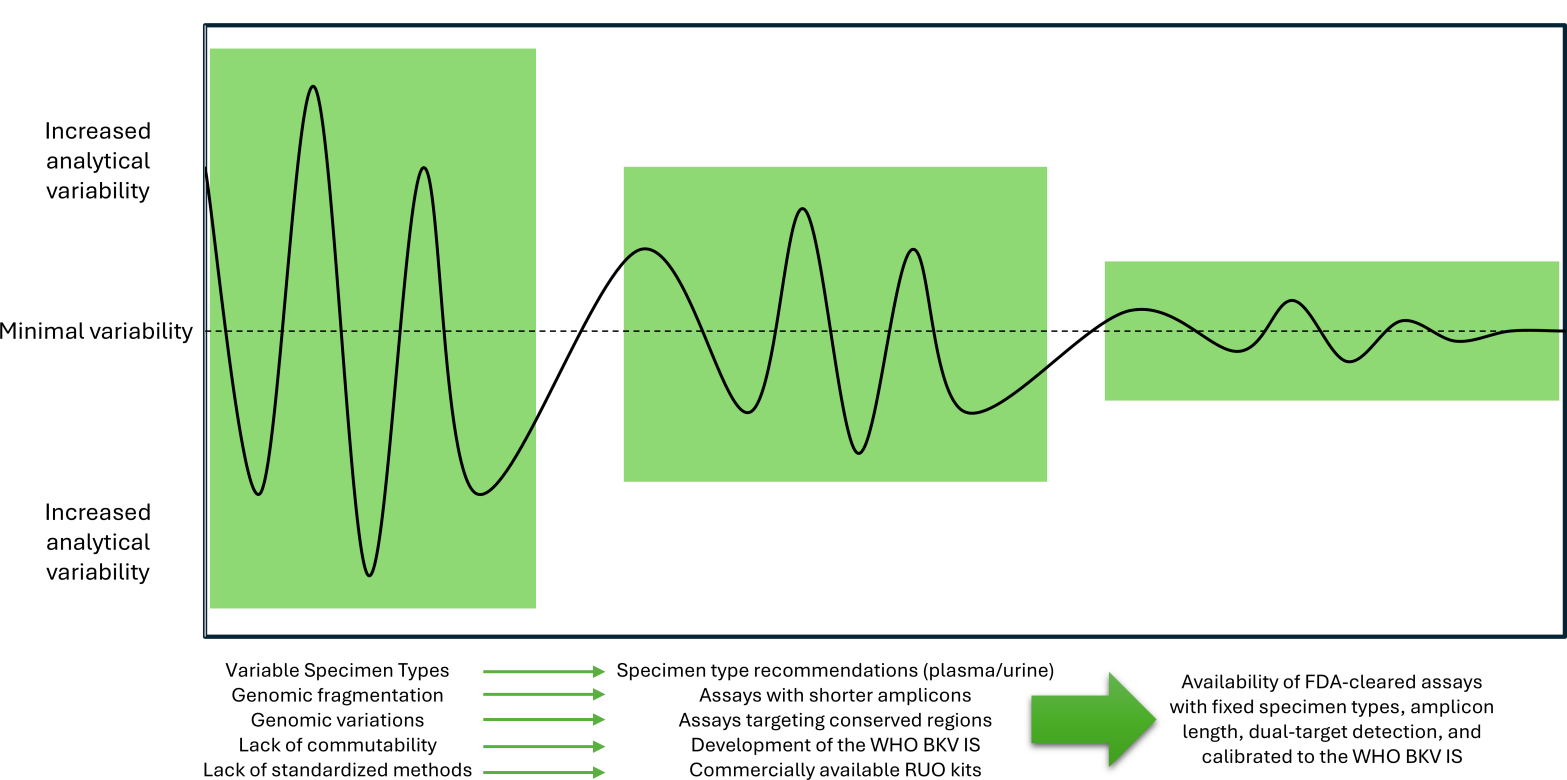
Modeling impacts of laboratory effects on BKV analytical variability. Historically, several factors have contributed to the variability observed in BKV analytical measurements. As testing has evolved, the introduction of guidelines, standard materials, and commercially available solutions has decreased this variability. The emergence of optimized FDA-cleared methods to mitigate the identified sources of variability reduced their effects even further. WHO BKV IS, World Health Organization BK Polyomavirus International Standard; RUO, Research Use Only; FDA, U.S. Food and Drug Administration.

A standardized BKV assay that is commercially available is an important achievement toward the harmonization of BKV clinical guidelines that prescribe actions based on strictly defined thresholds. If additional transplant centers were to adopt common commercially available BKV assays in routine monitoring of both renal transplant and HSCT recipients, the effects of disparate specimen types, fragmentation, commutability, calibration, extraction and purification, and sequence variations could be dramatically reduced (Fig. 1). However, the reliance on a single manufacturer to provide this testing comes with some uncertainty. First, the cost to obtain instrumentation for testing that is already performed can be prohibitive for some laboratories. Even if current LDTs exhibit variability, clinicians ordering testing may be fully aware of this possibility and incorporate that uncertainty into their practice accordingly, through either more frequent or repeat testing of potentially actionable results. Asking a laboratory to switch to a method that is unfamiliar to clinicians may generate unnecessary anxiety without an obvious benefit. Second, we have all become keenly aware that supply-chain constraints, often through no fault of the manufacturer, can limit access to required reagents. This could occur through lack of raw materials to build the *in vitro* diagnostic assay or worse, if an FDA recall directs the immediate cessation of testing. Moreover, the impact of regulation of LDTs cannot be ignored. The FDA’s final rule mandates that laboratories implement FDA-cleared testing options, should one exist, in favor of LDTs, regardless of the cost to the lab (50). Exemptions are offered, but over time will ultimately lead to the disappearance of most LDTs for BKV monitoring. Without the availability of additional FDA-cleared assays, laboratories risk reliance on a single manufacturer to provide the needed testing resources. Thus, diversification of FDA-cleared methods using different target sites can hopefully mitigate the effect of viral sequence changes that plagued FDA-Emergency Use Assays during the COVID-19 pandemic (51). To further illustrate this possibility, a discrepant plasma sample was recently identified during a comparison between the FDA-cleared cobas assay and an LDT (52). The authors could not resolve the discrepancy as sequence analysis was not performed, but they hypothesized that sequence differences in primer binding sites were responsible for failure of the cobas assay to detect a comparable titer. In the absence of alternate methods to mitigate assay results inconsistent with the clinical picture, there is a possibility that rare patients with presumptive BKVAN may go unrecognized until the damage is irreversible.

In summary, a standardized commercially available assay for monitoring patients for BKV infection represents an important resource in the laboratory’s diagnostic arsenal. Yet one must wield this tool with deference to known biological, analytical, and clinical limitations.
